# Some Incidents of Practice in Mexico

**Published:** 1889-09

**Authors:** N. H. Wheeler

**Affiliations:** Guanajuato.


ARTICLE VIII.

### SOME INCIDENTS OF PRACTICE IN MEXICO.

BY DR. N. H. WHEELER, OF GUANAJUATO.

There is no question but what these people have been horribly treated in the extraction of teeth. The key of Garengeot or key instrument has been in use for years and is still used to a great extent by the barbers, who are the usual surgical dentists throughout the country, and as a consequence they are afraid to have anything done to their teeth. Nearly three years ago in Lerdo, a gentleman living near there was suffering from pain in a lower molar, left side, the first and third molars had been extracted; he was advised to send for me but instead sent for a barber with his key. He applied to me a few days after suffering greatly from pain in his jaw. On examining, I found the bone had been split from the socket of the first molar to the third on the inside but was not separated. Hoping that it would reunite I pressed it to its place and applied a soothing lotion on a layer of cotton which soon easied the pain; gave instructions to continue it and suppose it resulted in a complete cure as he did not return.

I have a lower molar forcep that has a history. A year and a half ago while in Santiago Papasquiaro I bought them and then learned their history. A young fellow had purchased them sometime before with the expectation of relieving suffering humanity; his results were far beyond his expectations. His first and only patient was a man who desired a lower molar extracted. The operation was undoubtedly original, he took a piece of wood and placed it across the teeth in front of the one to be drawn, then taking the forceps he secured a good hold of the tooth, a simple question of mechanics—tooth weight to be moved, wood fulcrum, forceps lever, jaw to sustain the weight, something must move. The young man was confident, he applied the weight to the lever, result the jaw broke both sides. A week after the patient was buried, inflammation having set in. The young man sold the forceps and quit.

They have a superstition that to extract an upper molar you will pull out the eye at the same time and will tell you that so-and-so had a tooth pulled and he lost his sight, he probably did afterwards, but that that was the direct cause no one would believe for an instant, except bad treatment.

